# In Silico Methods for the Discovery of Orthosteric GABA_B_ Receptor Compounds

**DOI:** 10.3390/molecules24050935

**Published:** 2019-03-07

**Authors:** Linn M. Evenseth, Dawid Warszycki, Andrzej J. Bojarski, Mari Gabrielsen, Ingebrigt Sylte

**Affiliations:** 1 Molecular Pharmacology and Toxicology, Department of Medical Biology, Faculty of Health Sciences, UiT—The Arctic University of Norway, NO-9037 Tromsø, Norway; linn.evenseth@uit.no (L.M.E.); mari.gabrielsen@uit.no (M.G.); 2 Department of Medicinal Chemistry, Institute of Pharmacology, Polish Academy of Science, Smetna 12, 31-343 Kraków, Poland; warszyc@if-pan.krakow.pl (D.W.); bojarski@if-pan.krakow.pl (A.J.B.)

**Keywords:** GABA_B_ receptor, orthosteric binding site, virtual screening, ligand-based screening, structure-based screening

## Abstract

The GABA_B_ receptor (GABA_B_-R) is a heterodimeric class C G protein-coupled receptor comprised of the GABA_B1a/b_ and GABA_B2_ subunits. The endogenous orthosteric agonist γ-amino-butyric acid (GABA) binds within the extracellular Venus flytrap (VFT) domain of the GABA_B1a/b_ subunit. The receptor is associated with numerous neurological and neuropsychiatric disorders including learning and memory deficits, depression and anxiety, addiction and epilepsy, and is an interesting target for new drug development. Ligand- and structure-based virtual screening (VS) are used to identify hits in preclinical drug discovery. In the present study, we have evaluated classical ligand-based in silico methods, fingerprinting and pharmacophore mapping and structure-based in silico methods, structure-based pharmacophores, docking and scoring, and linear interaction approximation (LIA) for their aptitude to identify orthosteric GABA_B_-R compounds. Our results show that the limited number of active compounds and their high structural similarity complicate the use of ligand-based methods. However, by combining ligand-based methods with different structure-based methods active compounds were identified in front of DUDE-E decoys and the number of false positives was reduced, indicating that novel orthosteric GABA_B_-R compounds may be identified by a combination of ligand-based and structure-based in silico methods.

## 1. Introduction

Virtual screening (VS) is the application of knowledge-based computational methods to identify novel compounds [1]. VS methods are divided into two major categories: ligand-based drug discovery (LBDD) methods and structure-based drug discovery (SBDD) methods [2]. The LBDD methods use information about known ligands (e.g., structure, target affinity/activity and physico-chemical properties) to search for new compounds, while the SBDD methods use structural information about the drug target and ligand-target complexes. LBDD and SBDD are time- and cost-effective methods that either alone or in combination have led to the discovery of novel compounds towards assorted targets, including the α1a adrenergic receptor, the serotonin transporter and the 5-HT_7_ receptor [3,4,5,6].

γ-Aminobutyric acid (GABA) is the main inhibitory neurotransmitter in the mammalian central nervous system (CNS). GABA exerts its physiological effects by binding to the ionotropic GABA_A_ and GABA_C_ receptors and the metabotropic GABA_B_ receptor (GABA_B_-R) [7]. The GABA_B_-R is an obligate heterodimeric assembly, comprised of the GABA_B1a/b_ and GABA_B2_ subunits, that belongs to class C of G-protein coupled receptors (GPCRs) [8,9]. Each subunit contains an extracellular domain called the “Venus flytrap” (VFT) domain, and a heptahelical transmembrane (7TM) domain. The VFTs have a bi-lobular architecture with two distinct domains, Lobe 1 (LB1) and Lobe 2 (LB2), which come into close contact upon agonist binding, hence the name VFT [9]. The GABA_B1a/b_ is responsible for the ligand binding through the orthosteric site located in the VFT. The GABA_B2_ VFT does not bind to any known ligands, as shown by radiolabelled ligand binding and mutagenesis studies, but is important for the activation as the ectodomain interacts with the GABA_B1a/b_ ectodomain to enhance agonist affinity [10,11]. The transmembrane part of the GABA_B2_ subunit hosts an allosteric binding site and is responsible for G-protein coupling [12,13,14]. The three-dimensional (3D) structure of the entire GABA_B_-R is not known, however, eight X-ray crystal structures of the VFTs co-crystalized with different agonists or antagonists and one of the VFT apo-form have been published [9].

The GABA_B_-R is linked to a variety of neurological and neuropsychiatric disorders including memory and learning deficits, addiction, epilepsy, anxiety and depression, and is an interesting target for drug intervention [15,16,17,18]. However, at present, the agonist baclofen (β-(4-chloro-phenyl)GABA) is the only marketed drug targeting the GABA_B_-R. Baclofen is used as a muscle relaxant and antispastic agent to treat muscle spasticity and other muscle symptoms caused by e.g., multiple sclerosis [19,20]. A major drawback with baclofen is the side effects which include dizziness, nausea, insomnia, and hallucinations caused by abrupt withdrawal [21]. Animal models have also linked baclofen and other GABA_B_-R agonists to anti-addictive effects towards nicotine, cocaine and alcohol, however, clinical studies of baclofen in alcohol abuse have shown conflicting results [22,23,24,25]. Animal cognition models such as the swim-test and plus-maze test, have indicated that baclofen also has anxiolytic effects [18,26,27]. GABA_B_-R antagonists show antidepressant effects in different variants of the swim test [18,28], while baclofen show worsening of depression symptoms [29,30]. The newly discovered concept of ligand bias (ligand functional selectivity) emphasises the benefit of discovering new compounds that promote beneficial signalling pathways, while at the same time blocking potential deleterious GABA_B_-R pathways. New orthosteric compounds may expand the knowledge about the physiological importance and the activation mechanism of the receptor [31,32], and be interesting as drug or probe candidates, either alone or in combination with allosteric modulators.

At present, fewer than 15 antagonists and approximately 40 agonists are classified as active GABA_B_-R compounds [33]. Most of them are analogues of GABA or baclofen. Their low structural diversity may indicate that the conformational space of orthosteric GABA_B_-R compounds is not fully explored. In the present study, we have evaluated the classical LBDD methods, chemical fingerprinting and pharmacophore modelling, and the SBDD methods docking and scoring, structure-based pharmacophores (e-Pharmacophores) and linear interaction approximation (LIA) models for their predicative ability in VS. The aim was to identify a practical VS workflow for identification of orthosteric GABA_B_-R compounds. Our results suggested that large structural similarities between known compounds limits the feasibility of ligand-based in silico methods, but by combining ligand-based methods with structure-based in silico methods, novel orthosteric GABA_B_-R compounds may be identified, and the number of false positives may be reduced.

## 2. Results

### 2.1. Compound Datasets

All ChEMBL (version 24_1, EMBL-EBI, Hinxton, Cambridgeshire, United Kingdom) compounds tested for GABA_B_-R activity were downloaded and used to generate two datasets, one with high affinity/activity compounds hereafter called active compounds, and one with low affinity/activity and inactive compounds, hereafter called inactive compounds. Threshold values for being including in the set of active compounds were: IC_50_ < 4100 nM, K_i_ < 1500 nM, EC_50_ < 25 μM, or fold changes/inhibition indicating higher activity than GABA, and that the compound has been tested in assays of cloned or native human or rat GABA_B_-R. The IC_50_ value is defined as the half maximum inhibitory concentration, while the EC_50_ value is the concentration of a compound needed to produce half maximal response [34]. In total, 217 entities were downloaded, but after removal of duplicates the dataset of active compounds contained 55 compounds (13 antagonists and 42 agonists) (Supplementary Material, Table S1), while the inactive contained 97 compounds (Supplementary Material, Table S2).

The active compounds were structurally clustered into four clusters of agonists (cluster 2: 12 compounds, cluster 4: nine compounds, cluster 5: four compounds and cluster 6: 13 compounds), and one of antagonists (cluster 3: 11 compounds). In addition, two antagonists and four agonists not fitting into other clusters were grouped together in cluster 1. In the following, these compounds are termed outliers. A dataset of DUD-E decoys (assumed non-binders) were generated from the structure of the active compounds. Fifty DUD-E decoys were generated per compound, giving a total of 300 DUD-E decoys for the cluster of outliers (cluster 1), 1900 DUD-E decoys for agonists (cluster 2: 600, cluster 4: 450, cluster 5: 200 and cluster 6: 650) and 550 DUD-E decoys for antagonists (cluster 3).

### 2.2. Fingerprinting

For each cluster of active, molprint2D (M2D) chemical fingerprints were used to generate modal (average) fingerprints that were used to search the active/DUD-E decoy datasets. The Tanimoto similarity metric method was used to evaluate the results. The evaluation showed that the fingerprinting approach was not able to rank active compounds in front of DUD-E decoys (results not shown).

### 2.3. Development and Evaluation of Ligand-Based Pharmacophore Models

**Figure 1:**
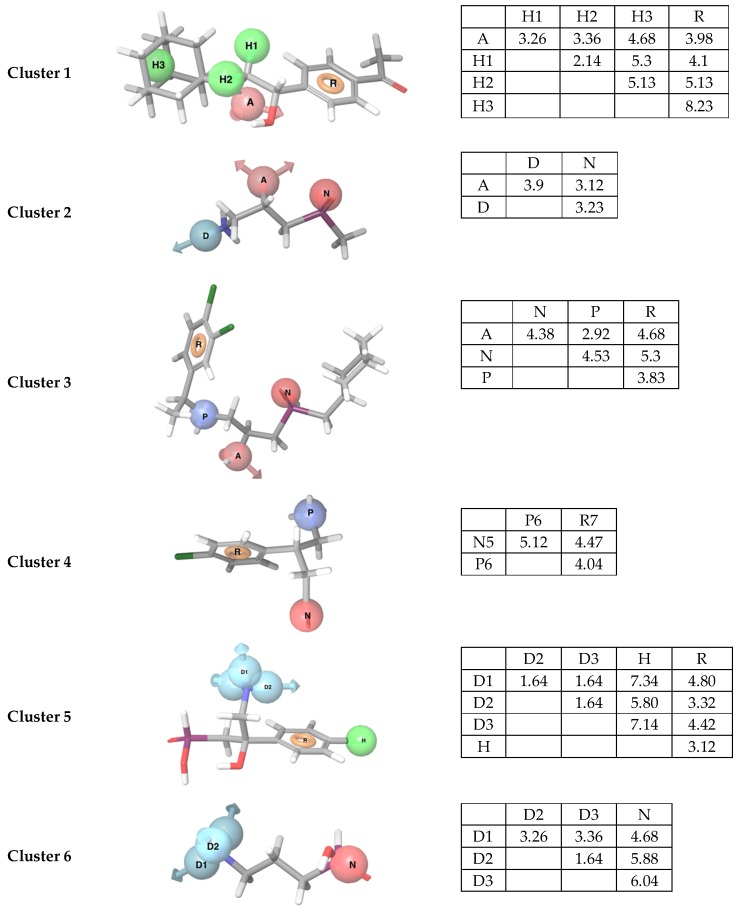
Pharmacophore models obtained from each cluster with the matrix of distances (Å) between features. The best mapped compound for cluster 1–6, CHEMBL2322934, CHEMBL113348, CGP54626, baclofen, 27 and CHEMBL112203, respectively, are displayed. Feature abbreviations; hydrophobic feature: H, hydrogen bond acceptor feature: A, hydrogen bond donor feature: D, aromatic feature: R, positively charged feature; P, and negatively charged feature: N.

Pharmacophore models were evaluated by mapping the compound datasets of active, inactive and DUD-E decoys to the hypotheses. One pharmacophore model per cluster was selected based on the Matthews correlation coefficient (MCC) and the “Goodness of Hits” score (GH). All pharmacophore hypotheses contained three to five features (Table 1, Figure 1). The statistical evaluation displayed variation in the quality of the generated pharmacophores with a range of the MCC and GH values from 0.20 to 0.95 (Table 1). The model giving lowest GH and MCC scores was generated from the structural cluster containing outliers. In total, 23 of 650 DUD-E decoys generated for GABA_B_-R antagonists and 115 of 2100 DUD-E decoys generated for agonists were found to be false positives by the pharmacophore mapping. Mapping all 55 actives to the agonist-based models showed that the models not only recognized agonists, but also some of the antagonists. In addition, all agonist based models identified agonists in other clusters. The more general models with few features, like those of cluster 4 and 6, identified most compounds (Table 1). The antagonist-based model identified only antagonists. Mapping of the 97 inactive compounds showed that the pharmacophore models recognized 61 of the compounds in the inactive dataset.

### 2.4. Development and Evaluation of Structure-Based e-Pharmacophore Models

**Figure 2:**
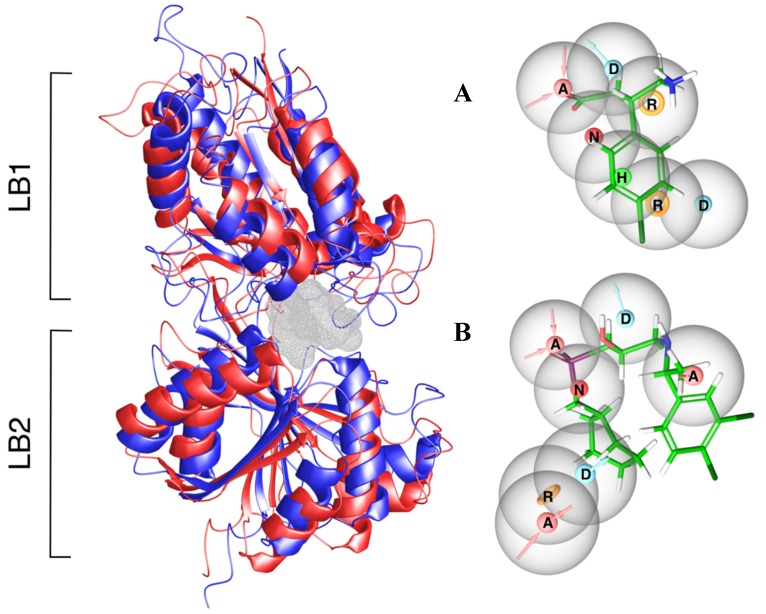
The X-ray crystal structure of the GABA_B_-R VFT superimposed in the active (blue) and inactive (red) conformation with the binding pocket cavity showed in grey mesh. (**A**) The e-Pharmacophore of the agonist-induced active VFT conformation displayed with baclofen (**B**) The e-Pharmacophore of the inactive antagonist-induced VFT conformation with the antagonist CGP54626. Both e-Pharmacophores are shown in the same view corresponding to their orientation in the binding pocket cavity (left) with LB1 up and LB2 down. Feature abbreviations; hydrophobic feature: H, hydrogen bond acceptor feature: A, hydrogen bond donor feature: D, aromatic feature: R, positively charged feature: P, and negatively charged feature: N.

Structure-based pharmacophore models (e-Pharmacophores) for an agonist-induced VFT conformation (Figure 2A) and antagonist-induced VFT conformation (Figure 2B) were generated using the Phase program [35]. A library of 441 unique fragments were mapped to the binding pocket of the antagonist-induced (inactive) VFT conformation (PDB ID: 4MR7) and the agonist-induced (active) VFT conformation (PDB ID: 4MS4).

**Figure 3:**
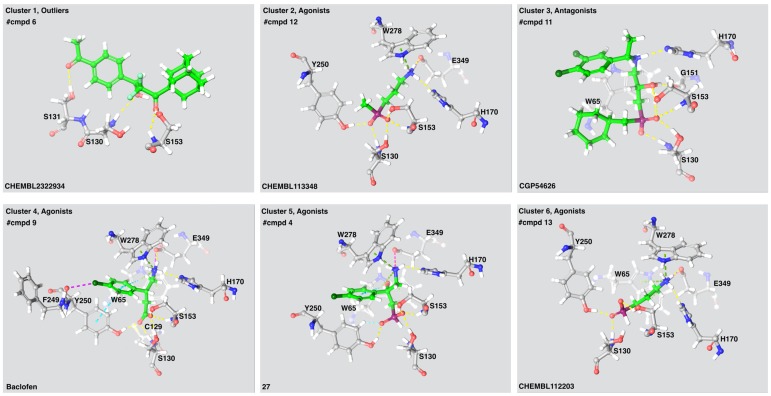
The ligand-receptor interactions of a selected compound from each cluster. Compound name is indicated in the lower left corner of each panel. The number of compounds (#cmpds) in each cluster and the intrinsic activity of the compounds is indicated. Cluster 1 consist of two antagonists and four agonists. Yellow lines: hydrogen bonds, green lines: Pi-cation interactions, Cyan lines: salt bridges, light blue lines: aromatic hydrogen bonds, magenta—halogen bond.

Mapping the fragment library in the inactive VFT conformation identified an aromatic feature in LB2 close to hydrophobic- and aromatic residues (Tyr250 and Trp278), together with a hydrogen bond donor and a hydrogen bond acceptor feature (Figure 2B and Figure 3). In LB1, one hydrogen bond donor, one hydrogen bond acceptor and one negative charged feature were identified. These features were connected to Cys129 and the serine residues located in position 130, 131, 152, 153 and 154. Some of these serine residues were involved in both agonist and antagonist binding (Figure 2 and Figure 3) as described by Geng et al. [9].

The e-Pharmacophore features in the agonist-induced VFT were clustered closer together than those of the antagonist-induced, with shorter distances between features (Figure 2). An aromatic group was located close to the Tyr279 and Trp278 in LB2 and Tyr250 in LB2. A hydrophobic feature was located in LB2 in close proximity to the hydrophobic part with aromatic residues almost buried inside the VFT. A hydrogen bond donor was also located in the cleft between LB1 and LB2, almost at the opening of the VFT. Another aromatic ring was located between LB1 and LB2 in close proximity to Trp278, Trp65 and His170. One hydrogen bond donor and one acceptor were in LB1 close to the Ser152 and Ser153 as for the inactive VFT conformation (Figure 2 and Figure 3).

Mapping the datasets of active compounds and DUD-E decoys to the e-Pharmacophore models showed that the e-Pharmacophore features were not selective for active compounds. In the antagonist-induced conformation four of total 13 unique antagonists and 602 DUD-E decoys were mapped, while in the agonist-induced conformation only nine out 42 agonists and 1000 DUD-E decoys (the maximum number) were mapped.

### 2.5. Analysis of the Docking Results

#### 2.5.1. Docking of Active GABA_B_-R Compounds

The dataset of 55 GABA_B_-R active compounds were docked in both agonist (PDB ID: 4MS4) and antagonist (PDB ID: 4MR7) induced VFT conformation. The Cα Root Mean Square deviation (RMSD) between these conformations was 7.0 Å, with largest differences in loop regions. The overall RMSD of residues within 5 Å of the co-crystalized ligands was 2.1 Å. Superimposition showed that the active conformation had a more closed VFT than the inactive conformation (Figure 2).

The average docking score of the 42 agonists in the agonist-induced conformation was −8.3 kcal/mol. The best score was −11.2 kcal/mol, while the poorest was −5.7 kcal/mol. Docking antagonists into the agonist-induced VFT gave an average docking score of −5.9 kcal/mol, and with poses inconsistent with X-ray structure complexes. The average docking score for the 13 antagonists in the antagonist-induced VFT was −7.1 kcal/mol, where the best score was −8 kcal/mol and the poorest −5.6 kcal/mol. Docking of the agonists into the antagonist-induced VFT gave an average docking score of −6.4 kcal/mol.

Ser130 and Ser153 interacted with all 55 active compounds, independent of intrinsic activity. The agonists were fully buried within the receptor interior, inaccessible to solvent, thereby increasing the number of interactions between the agonists and the receptor and stabilizing the closed VFT conformation. As known agonists and most antagonists are analogues of GABA or baclofen, all ligands selected as cluster representatives showed similar ligand-interaction patterns (Figure 3). The LB1 residues Ser130 and Ser153 formed hydrogen bonds with a carboxylic acid moiety present in all agonists and antagonists. The LB1 residue Tyr65 and the LB2 residue Tyr250 stabilized the agonists by forming π-stacks or π-cation interactions, while Glu349 formed a salt bridge or ionic interaction with the amine moiety present in the agonists (Figure 3). The LB2 residue Trp278 formed a π-cation interaction with the ligands selected from three of four agonist clusters. Interactions between antagonists and hydrophobic residues in LB2 such as Trp278 and Tyr250 were observed for the highest affinity antagonists. The GABA_B_-R antagonists are bigger and more bulky than agonists and will most likely prohibit the VFT closing as previously described by Geng et al. [9].

The average docking scores of known agonists and antagonists (dataset of active compounds) were used as threshold values for evaluating the docking of inactive compounds and false positive DUD-E decoys from the pharmacophore mapping.

#### 2.5.2. Docking of Inactive GABA_B_-R Compounds

Docking the dataset of 97 inactive GABA_R_-R compounds (Supplementary, Table S2) showed that 79 of 97 compounds docked into the agonist-induced VFT, while all of them could dock into the inactive antagonist-induced conformation. In total, 13 compounds scored better than the threshold for agonists (average score of the 42 agonists) in the agonist-induced conformation. The compounds scoring higher than threshold are baclofen analogues containing an aromatic ring with a halogen and an alkyl chain with amino and carboxylic end groups. In total, 10 from the set of inactive compounds scored better than the threshold for antagonists (average score of the 13 antagonists) in the open inactive antagonist-induced VFT conformation.

#### 2.5.3. Docking of False Positive Compounds from the Ligand-Based Pharmacophore Mapping

The false positive DUD-E decoys from the pharmacophore mapping were docked into the X-ray crystal structures in order to reveal if a succeeding docking procedure could reduce the number of false positives in a VS campaign. Twenty-three of the 650 DUD-E decoys generated from antagonists were identified as false positives by pharmacophore mapping. All of them scored worse than the threshold value for known antagonists (−7.1 kcal/mol), and 11 scored better than the poorest scored known antagonist (−5.6 kcal/mol). As the average docking score was used as the threshold, none was applied for further investigation by LIA models.

In total 116 DUD-E decoys generated from agonists were found to be false positive after pharmacophore mapping. Of these, five had a docking score better than the agonist threshold (−8.0 kcal/mol) and 72 out of the 116 gave a better score than the poorest scored agonist (−5.7 kcal/mol). The five compounds with docking score better than threshold were studied in the agonist LIA model to evaluate if the LIA method could identify the compounds as theoretically inactive DUD-E decoys.

### 2.6. Generation and Evaluation of LIA Models

The Liaison software in combination with Strike included in the Schrödinger package [36,37] were used for generating linear interaction approximation (LIA) models of agonists and antagonists and predicting ligand-receptor affinities using the LIA models. A training set of 11 agonists were used to construct the agonist LIA model, while a training set of eight antagonists were used to generate the antagonist LIA model. The models were evaluated by true positives from the pharmacophore mapping, but excluding those included in the training sets.

**Figure 4:**
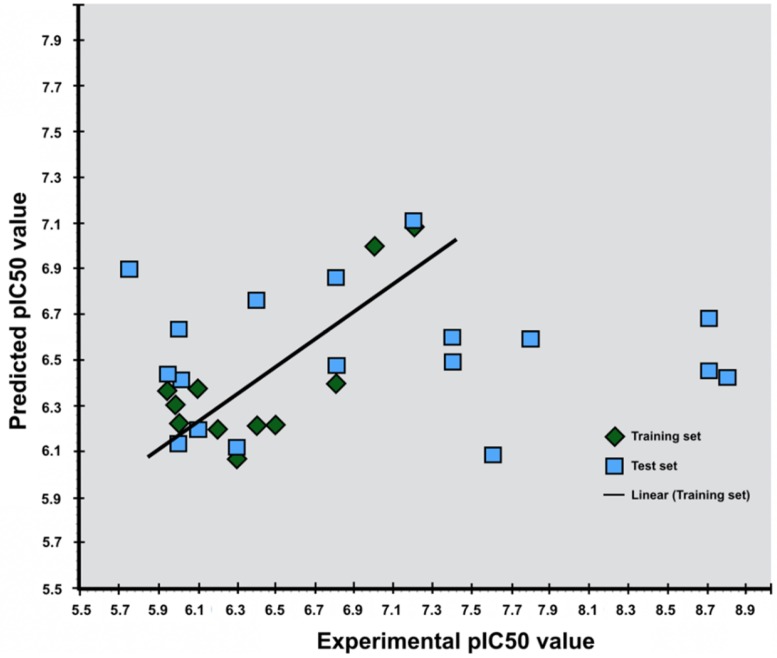

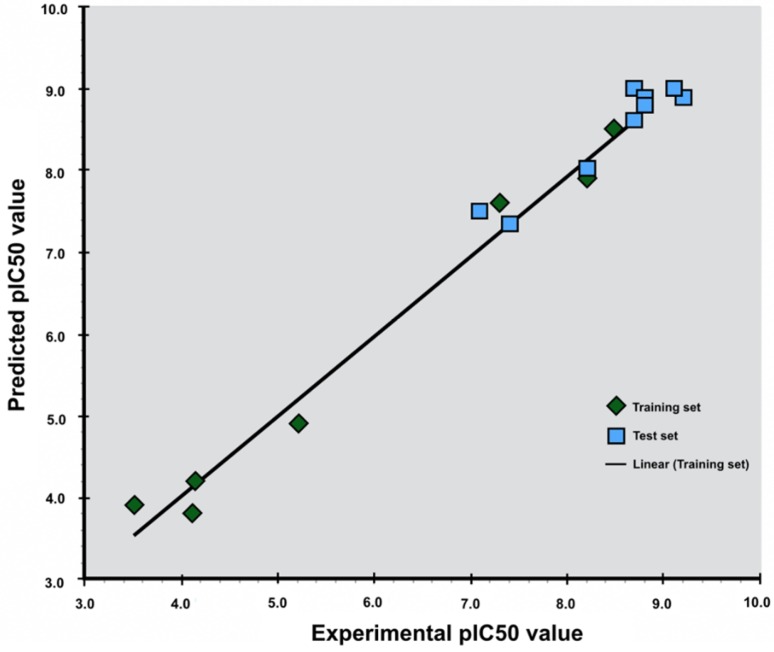
Scatter diagram of experimental and predicted pIC_50_ values using the LIA method for agonists (**top**) and antagonists (**below**). The green squares are the ligands of the training set used to generate the parameters for predicting pIC_50_ value of the ligands of the test set (blue squares).

The LIA model generated for antagonists gave a R^2^ value of 0.98 indicating that the predicted pIC_50_ values highly correlate to the fitted regression line of the experimental pIC_50_ values. The standard deviation was 0.41 with a P-value of 0.0044 (Table 2 and Figure 4). The LIA model generated for agonists gave a R^2^ value of 0.61, which indicates that the predicted pIC_50_ values correlate to the fitted regression line of the experimental pIC_50_ values. The standard deviation was calculated to be 0.32 with a *p*-value of 0.074 (Table 2), and applying the LIA model to predict the pIC_50_ values of the true positive from the pharmacophore screening, gave less accurate results for agonists then for antagonists (Figure 4).

The agonist LIA model was applied to the five false positive DUD-E decoys from docking. Only one out of the false positives had a predicted pIC_50_ value < 5. Five is normally considered as the threshold pIC_50_ value for activity, and the agonist LIA model could therefore identify only one of the five compounds as a false positive.

## 3. Discussion

The GABA_B_-R has a large potential as a target for new drugs. The number of known compounds is limited and most of them are analogues of the endogenous compound GABA and the therapeutically used agonist baclofen. Known compounds represent a quite small conformational space that complicates the understanding of molecular descriptors contributing to differences in affinity and intrinsic activity, and it is a challenge to identify new and improved orthosteric GABA_B_-R compounds.

Our dataset of active compounds consists of compounds from experimental studies using different assay conditions (Supplementary Material, Table S1), which is a challenge for the robustness of the dataset since binding data from different assays are not necessary directly comparable. However, in order to get an acceptable number of compounds for the ligand-based approaches it was necessary to include compounds that had been evaluated using different experimental procedures. We used threshold values for experimental activity to discriminate between active and inactive compound datasets in order to reduce the influence of low affinity compounds on our in silico models. The dataset of inactive compounds therefore contained not only inactive compounds, but also compounds with low GABA_B_-R activity. The compounds with highest affinity in the set of inactive compounds were four antagonists also used to generate LIA models (Supplementary Material, Table S1). Other low affinity/activity compounds in the set of inactive compounds had activity values far below these antagonists. The low affinity compounds are also structural analogues of GABA and baclofen, which complicates the present study since the datasets of active and inactive compounds both contains GABA and baclofen analogues. Discriminating between the active and inactive datasets by the LBDD and SBDD in silico methods in the present study is therefore challenging.

### 3.1. Ligand-Based Screening

#### 3.1.1. Fingerprinting, Clustering and Modal Fingerprints

Average fingerprints for each cluster failed to recognise the compounds from which the fingerprints were generated in front of active compounds from other clusters, and in addition, they did not discriminate between actives and DUD-E decoys. This was not a surprise due to the structural similarities between clusters. Selecting more structurally divergent compounds was not possible. Using fingerprinting alone in a VS campaign for new orthosteric compounds would therefore most likely not identify novel GABA_B_-R compounds.

#### 3.1.2. Ligand-Based Pharmacophore Modelling

One pharmacophore hypothesis per cluster was selected after statistical evaluation (Table 1). Ideally, a higher number of pharmacophores would be preferable for screening to account for structural diversity, but this was not possible due to structural similarities between the clusters. The pharmacophores for cluster 1, 2 and 5 gave poor discrimination between actives and DUD-E decoys, and identified many false positive compounds. In these models, the hypothesis composition was very general with repetitive features able to align with multiple compound structures. Low discrimination and retrieval of many false positives is not necessarily negative in a VS workflow, as also these models could contribute to discovery of new structural scaffolds.

A pharmacophore model of only three features can be problematic as several compounds may fit to the model, and the models may select many false positives during VS. The GABA_B_-R agonists are small with few functional groups, which gives few pharmacophore features in the hypothesis. Changing the intersection distance constraints from 2 Å to 1.5 Å (for cluster 5 and 6) gave demanding hypotheses, i.e., an amine group with two hydrogen bond donating features, whereas the default intersection distance (2 Å) would generate only one hydrogen bond donor or preferably a positive charged site, as seen for two of the clusters. A main purpose of generating cluster-based pharmacophore models was to increase the possibility of retrieving new chemotypes. Decreasing the intersection distance to avoid repetitive feature composition as most of the actives contains a positive- and a negative charged group in the same positions, could also contribute to new chemotypes.

Known antagonists are larger than agonists, and may give a higher number of features in pharmacophores than agonists. However, there are only 13 known high affinity antagonists for the GABA_B_-R. Of these, 11 were grouped into the same structural cluster (cluster 3) when applying the similarity metric and thereby only one pharmacophore model was generated. In a VS approach this hypothesis would be considered as accurate due to selectivity towards active ligands, and it is not too strict in terms of feature composition.

Mapping the inactive set of compounds to the pharmacophores also confirms the high structural similarity between the active and inactive compound datasets (Supplementary, Tables S1 and S2) as 61 out of the 97 could be mapped to the models.

### 3.2. Structure-Based Screening

#### 3.2.1. Structure-Based e-Pharmacophore Models

E-Pharmacophores can be applied for VS and compound optimization (e.g., hit-to-lead and lead optimization). Using fragments that cover a wide range of functional groups to map the binding pocket gives new insight into the properties of the binding pocket. This knowledge can be used to guide ligand growing into areas of the pocket where specific ligand features are beneficial, as suggested for the areas discovered in the inactive VFT structure. This possibility can be more restricted when applying active ligands for e-Pharmacophore development, especially if the information about active ligands is limited, as for the GABA_B_-R.

The antagonist-based e-Pharmacophore identified an aromatic feature in the LB2 moiety close to Tyr250 and Tyr278. In both the X-ray complexes and our docking studies, interactions are seen only for high affinity antagonists at this site [9], indicating that this feature is important for high affinity antagonist binding. In addition, both a hydrogen bond donor and a hydrogen bond acceptor feature located in this area were unexplored in our docking studies, despite being within the generated grid map (Figure 2). These sites could be further explored by growing antagonists anchored in LB1 towards these points using fragments complementary to the discovered features. As described, amino acids in LB1 are essential anchoring points for antagonists and the features located in this site were not unexpected. In the LB1, a negatively charged feature, a hydrogen donor and one hydrogen bonding accepting feature were identified which represents serine residues that are necessary for both agonist and antagonist binding. None of the identified features in the agonist-induced active VFT conformation revealed any areas not already identified in our docking studies.

#### 3.2.2. Docking

Visual inspection of the GABA_B_-R VFT co-crystalized with agonists or antagonists showed that most of the α-acid groups formed interactions with residues in LB1 such as His170, Trp65, Ser130 and Glu349. Amino acid located in LB2 such as Tyr250 and Trp278 interact with the agonists in all complexes. Trp65 forms van der Waals interactions with all antagonists. An interesting observation was a ~180° flip of Trp278 in the structure co-crystalized with baclofen compared with the other agonist bound VFT 3D structures [9]. This flip is probably necessary for stabilizing the aromatic ring of baclofen. Visual inspection of selected inactive and low affinity compounds that scored better than threshold in both receptor conformations showed similar binding patterns as the active compounds, which also was expected due to structural similarities.

None of the 23 false positive antagonist-like DUD-E decoys from the pharmacophore mapping scored better than the average score of active antagonists when docking into the antagonist-induced conformation. In the agonist-induced VFT, the number of false positive agonist-like DUD-E decoys was reduced from 116 to five when using the average score of active agonists as threshold. This indicates that using average docking scores of active compounds as a threshold in combined ligand- and structure-based VS for orthosteric GABA_B_-R compounds may have filtered out most of the false positives from the pharmacophore mapping. Identification of ligands with high affinity is desirable and to ensure fulfilling this criterion, the threshold for evaluating docking pose should be set to the average value instead of the value of the poorest scored active ligand in a VS campaign. By using the average value as threshold, it is of course a possibility of overlooking putative compounds, but most probably high affinity compounds would be identified. Using average docking scores may also account for the inaccuracy obtained by assuming similar activity of all generated enantiomers of a compound when the active form(s) is/are not known (Section 4.1).

Only two of nine available VFT structures were used in our docking. Ideally, the ligands should have been docked into multiple VFT conformations in order to account for the structural flexibility of the binding process [38]. However, the available X-ray crystal structures of the VFT are very similar. The RMSD between the binding site residues of the agonist bound VFTs is 0.26 Å, while the corresponding average value between six antagonists’ bound is 0.27 Å. The overall Cα-RMSD between agonist-induced VFTs was 2 Å, while corresponding RMSDs between antagonist-induced VFTs were in the range of 0.75 to 2 Å. Visual investigation showed that the main differences were in regions other than the binding pocket, and available VFT X-ray crystal structures were therefore not encountered as conformational distinct.

The necessity of docking as a step in a VS protocol for identifying GABA_B_-R compounds is clearly shown by the present study, but also the difficulty to differentiate between very similar compounds as shown by the docking of the false positives from the ligand-based pharmacophore screening. Ligand-based methods are more time- and cost-effective than docking and scoring, and in VS for new GABA_B_-R compounds, ligand-based methods may remove some compounds in the library that definitely do not bind, before a docking step reduces the number of remaining false positives.

### 3.3. Generation and Evaluation of LIA Models

The methodology for predicting ligand-protein free energies by comparing the bound complex to the free ligand-receptor state using an explicit-solvent for building a model to predict/correlate binding free energies, was first suggested by Åqvist [39,40]. Their approach is computationally demanding as it uses molecular dynamic simulations with an explicit water model for sampling conformations. Using this approach for screening of a large number of compounds is therefore problematic. In the present study, we have therefore evaluated the LIA method, which generates thermodynamic averages by using minimization as sampling method for the different molecular systems instead of MD. In contrast to the original Linear Interaction Energy (LIE) method by Åqvist et al., we have also used an implicit water model to speed up the calculations.

Åqvist found that the coefficients α = 0.18 and β = 0.5 (given a charged ligand) were sufficient to give results in agreement with experimental values for several protein systems. Later others reported that the values could be changed and still make intuitively sense [41]. However, our simplifications may affect the accuracy of the method, and may create coefficients different from the Åqvist LIE method. When a full simulation is not performed, the displacement of the water molecules from the receptor and placement of ligand in the pocket that is partly hydrophobic, is not necessarily satisfying in terms of calculating the energy and/or entropy.

Some coefficients obtained in the present study have negative values (Table 2). When using the OPLS2005 force field it is considered as acceptable if the γ value is negative due to changes in the cavity term [42]. Negative α and β coefficients, indicates that the van der Waals (vdW) and electrostatic forces favours the unbound state, but as previously discussed, the background of the LIE theory does not correspond completely with the methodology applied for calculating the Liaison parameters. Alman et al. applied the method for calculating the binding affinity of podophyllotoxin analogous for tubulin using MD for sampling, and got negative α, β and γ coefficients, but a significant squared correlation coefficient (R^2^ = 0.73) [43].

The *p*-value of the LIA model for agonists was slightly higher than the normally accepted p-value (<0.05), but was tolerated due to the correlation coefficient and low standard deviation. The LIA model for predicting pIC_50_ values for antagonists, performed well with a correlation coefficient of 0.98. The agonists have in general a lower pIC_50_ value than antagonists, and the threshold must be set accordingly. Totally four DUD-E decoys generated from agonist structures were considered as actives after pIC_50_ predictions by the agonist LIA model. DUD-E decoys generated from cluster 1 of outliers were included in the decoy set of both agonists and antagonists, and may have contributed to a lower accuracy.

The statistics of the agonist LIA model were significantly less specific than the model for antagonists. The results for prediction of pIC_50_ value of false positives were therefore not unexpected, but it cannot be ruled out that these compounds are actually binders as they are only assumed non-binders with physicochemical properties resembling known binders. The assumption that enantiomers have identical experimental values may significantly affect the accuracy of the agonist LIA model. Stereochemistry plays a major role in target selectivity and pharmacokinetics. Chiral drugs can behave very differently in a system, which points out the inaccuracy with assuming an identical pIC_50_ for enantiomers [44,45].

## 4. Materials and Methods

### 4.1. Datasets

All ChEMBL (version 24_1) compounds tested for GABA_B_-R binding were downloaded and used to generate compound sets of active and inactive compounds. The activity threshold values for being including in the set of active compounds were: IC_50_ < 4100 nM, K_i_ < 1500 nM, EC_50_ < 25 μM, or fold changes/inhibition indicating higher activity than GABA, and that they have been tested on assays of cloned or native human or rat GABA_B_-R. The dataset of actives contained 13 antagonists and 42 agonists, including enantiomers (assuming same activity measurement for enantiomers when not specified) [33,46,47,48,49,50]. MOLPRINT 2D (M2D) chemical fingerprints of all 55 active compounds were generated, before Hierarchical clustering using Tanimoto similarity matrix was performed in Canvas [51]. The number of clusters was set to 10, but after manual modifications and merging of singletons and doubletons, the total number of clusters was reduced to six (Supplementary, Table S1). The clustering method separated agonists from the antagonists, giving 1 cluster with antagonists and 4 clusters with agonists, in addition a cluster of outliers that merged singletons and doubletons from the initial clustering (4 agonists and 2 antagonists). The DUD-E methodology [52] was used to generate DUD-E decoys, using the structure of active compounds (agonists and antagonists) as input. Fifty DUD-E decoys per active ligand were generated. The compound structures were prepared by LigPrep [53] at a pH of 7.4. Tautomers were generated and the specified chirality of compounds was retained.

#### Phase Databases

Phase is an engine that is used in pharmacophore modelling [35]. The engine can also be used to generate and modify databases. A Phase databases were generated for each cluster of agonists and antagonist. In addition, a Phase database containing all 55 actives, and two DUD-E decoys databases one containing agonist-like decoys and one containing antagonist-like decoys were also generated. Default settings with generation of up to 50 conformers per ligand were used.

### 4.2. Ligand-Based Methods

#### 4.2.1. Fingerprinting

Modal fingerprints for each cluster were generated by averaging the M2D fingerprints of ligands in each cluster into cluster based modal fingerprints, representing each of the six clusters [54]. The fingerprints selectivity were evaluated by their ability to identify true actives from DUD-E decoys by Tanimoto similarity metric.

#### 4.2.2. Ligand-Based Pharmacophore Modelling

Pharmacophore models for each cluster of active compounds were generated in Schrödinger’s Phase [55] using all compounds in each cluster as input. The pharmacophore models were generated with default parameters: 10 conformers per rotatable bond, maximum 100 conformers per compound, 2 Å RMSD tolerance level for match [55,56]. As the compounds are very similar, the intersite distance for cluster 5 and 6, i.e., the distances between pairs of potential features in the pharmacophore composition were changed from default 2 Å to 1.5 Å to produce more variable hypotheses, including additional features than by default (Table 2). Selection of pharmacophore features was conducted automatically. Any manual selection of features (or constraints) [57] was not applied due to the limited number of active compounds available. A pharmacophore model was considered valid when the model mapped and matched at least 50% of the compounds used to generate that particular pharmacophore model. Mapping and matching were performed by representing each feature of a pharmacophore composition as a distance vector that must overlap with that of the mapped ligand in order to be considered as a match. The pharmacophore hypothesis from each cluster were also evaluated by mapping their respective database of DUD-E decoys generated by the DUD-E methodology [58].

After mapping the respective databases of DUD-E decoys and actives to the pharmacophore models, the accuracy of the models was evaluated by calculating the Matthews correlation coefficient (MCC) (Equation (1)) and “Goodness of Hits” score (GH) (Equation (2)). MCC and GH are calculated from the number of true positives (TP), false positives (FP), true negatives (TN) and false negatives (FN). The compound had to match all features specified for a model to be classified as active, with the exception of the cluster with outliners where the threshold was set to match four out of five features:

(1) $$\mathrm{MCC}=\frac{\mathrm{TP}\cdot \mathrm{TN}-\mathrm{FP}\cdot \mathrm{FN}}{\sqrt{\left(\mathrm{TP}+\mathrm{FP}\right)\left(\mathrm{TP}+\mathrm{FN}\right)\left(\mathrm{TN}+\mathrm{FP}\right)\left(\mathrm{TN}+\mathrm{FN}\right)}}$$

(2) $$\mathrm{GH}=\left(\frac{\mathrm{Ha}\left(3A+\mathrm{Ht}\right)}{4\mathrm{HtA}}\right)\cdot \left(1-\frac{\mathrm{Ht}-\mathrm{Ha}}{D}\right)$$

where Ha = TN, Ht = TP + FP, A = TP + FN and D = DUD-E decoys.

The last step in the evaluation, was mapping all active and inactive compounds across clusters to all generated models. MCC gives a correlation between the observed and predicted classifications, in this case actual active and false positive compounds. MCC can be used even if the number of compounds in each class differ [59]. The value can range from −1 to 1, where 1 represents a perfect prediction, 0 indicates a random prediction and −1 represents an inverse prediction [60]. GH scoring function takes into account the sensitivity, specificity and enrichment. The GH scoring thereby gives a good indication of model quality by compromising the yield and actives retrieved and by taking into account the hit list size in comparison with the library size. The score ranges from 0 to 1, where a score of 1 represents the ideal model that perfectly separates active and inactive compounds [61].

### 4.3. Structure-Based Methods

#### 4.3.1. Protein Preparation

Two crystal structure complexes of GABA_B_-R VFT with agonists (PDB IDs: 4MS3, 4MS4) and six with antagonists (PDB IDs: 4MR7, 4MR8, 4MR9, 4MS1, 4MRM, 4MQF) are present in the PDB-database. The PBD ID 4MR7 in complex with the antagonist CGP54626 with a resolution of 2.15 Å, and the PDB ID 4MS4 in complex with the agonist baclofen with a resolution of 1.9 Å were used for the structure-based studies. They were selected to represent the active agonist and inactive antagonist-induced VFT structures due to the resolution. The structures were pre-processed in Schrödinger Protein Preparation wizard with default settings; Hydrogen bonds were assigned with a PROPKA pH of 7. A restrained minimization was performed with converging heavy atoms at RMSD of 0.3 Å [62].

#### 4.3.2. Structure-Based e-Pharmacophore Model

The Phase program [35] was used to generate e-Pharmacophores. Receptor binding sites were defined using the centroids of residues involved in ligand binding in all agonist-receptor complexes and most of the antagonist-receptor complexes. For agonists the centroid of Tyr250, Ser130, Ser153, Glu349 and Trp278 was used, while for antagonists the centroid of Ser130, Ser153, Tyr65, His170, Gly151 and Tyr250 was used. The features of each pocket was then found by mapping a library of 441 unique fragments [35] to the binding pocket. The library consisted of 1–7 ionization/tautomer variants of each fragment, and each fragment contained 6–37 atoms with molecular weight ranging from 32 to 226 Da. In total, the set consisted of 667 low energy fragments with ionic and tautomeric states and with metal state penalties for each fragment. The fragments were docked into both X-ray crystal structures by using the Glide XP docking protocol, before the pose viewer file was used to generate e-Pharmacophores. The maximum number of features was set to seven and the hydrogen bond donors were projected as points instead of the default vectors.

The models were screened against the generated databases of active and DUD-E decoys in order to evaluate the capability of the models to select the active from DUD-E decoys. The matching rate of features was set such that at least four out of the seven features should match for the antagonists and three out of seven for agonists. The maximum number of hits was kept at the default number of 1000. The results were evaluated by calculating the Matthews correlation coefficient (MCC) and “Goodness of Hits” score (GH) (Equations (1) and (2))).

#### 4.3.3. Docking Studies

The docking was performed with the Glide program of the Schrödinger suit [63]. One grid map was generated per selected crystal structures, 4MS4 and 4MR7, by selecting the co-crystalized ligand as the centroid of the grid box. However, the grid size was increased by changing the inner box diameter from 10 Å to 15 Å such that larger compounds than the co-crystallized ligands could be docked. The remaining settings for the grid generation were kept at default values. A standard precision (SP) docking protocol in Glide was set up with enhanced sampling and generation of maximum 10 poses per ligand. Binding poses with Coulomb and van der Waals forces > 0 kcal/mol were by default filtered away, while ligand poses with RMSD < 0.5 Å were treated as duplicates and one of them was removed. The scoring threshold for agonists and the antagonists were found by calculating the average docking score of 42 agonists and the 13 antagonists, respectively. A cross-docking where agonists were docked into in antagonist-induced VFT conformation and antagonists into the agonist-induced was also performed.

After the docking, one representative compound from each cluster was selected for analysis and description of the interaction patterns between known orthosteric GABA_B_-R compounds and the VFT. The interactions between the selected ligands and the VFT were compared to identify potential differences in binding modes between the clusters and between agonists and antagonists. Cluster 1 was not included since it contains outliers of both agonists and antagonists. Known inactive and low activity compounds (compounds with IC_50_, K_i_ or EC_50_ values higher than those used in the selection of actives) were also docked and scored in the VFT of both agonist and antagonist-induced VFT conformation (Supplementary, Table S2).

The false positive compounds from the pharmacophore screening were also docked using the standard precision (SP) docking protocol in Glide with the same settings as previously described, to evaluate if the docking could correctly assign these compounds as TN in contrast to the pharmacophore screening which predicted these compounds as active.

### 4.4. LIA Model Development and Evaluation

In LIA calculations, molecular mechanics (MM) simulations are used to calculate energy of ligand both in a bound and unbound state, using a continuum solvation model. The Liaison program used the following equation to predict the free energy of binding (ΔGbind):

(3) ΔGbind=α(〈Ubvdw〉−〈Ufvdw〉)+β(〈Ubelec〉−〈Ufelec〉)+γ(〈Ubcav〉−〈Ufcav〉)

The brackets indicate that the calculation uses the average energy of conformations sampled during MM simulations. Uf describes the molecule free in solution and Ub describes the target-ligand complex. The energy terms are the van der Waals interactions (Uvdw), the electrostatic interactions (Uelec) and the cavity parameter (Ucav). A training set of compounds with known affinity is used to build an energy model by fitting three coefficients (α, β, and γ,) to their experimental free energy of binding. The models can then be used to predict affinities of ligands with unknown experimental affinity.

In total, 42 compounds (including enantiomers) were considered as highly active GABA_B_-R agonists. Their experimental values were converted to IC_50_ (assuming IC_50_ = K_i_ × 2) and then to pIC_50_ [64]. Six agonists were excluded since their experimental values were incompatible with conversion to pIC_50_ (Supplementary Material, Table S1). Without considering enantiomers, there were then 20 unique agonists and due to the low diversity in pIC_50_ totally 11 out of the 20 compounds were included in the training set for generating the agonist LIA model (Supplementary Material, Table S1). The remaining nine compounds were used in the test set in addition to all true positives identified by the pharmacophore mapping, however, agonists used in the training set were removed but enantiomers of these were kept.

Based on the affinity values, 13 compounds were considered as highly active GABA_B_-R antagonists. Three of these were selected for the training set, in addition 4 low affinity antagonists from the set of inactive were included in the training set (Supplementary Material, Table S1), to increase the structural diversity and the range of the pIC_50_ value to give more useful LIA models for identification of antagonists in a VS approach. Two of the included low affinity antagonists were from the X-ray complexes 4MQF and 4MRM of the GABA_B_-R VFTs. The test set consisted of true positives from the pharmacophore mapping which included eight of 10 remaining high affinity antagonists. Two of the high affinity antagonists were found to be false negatives as they were not retrieved by the pharmacophore mapping and therefor excluded from the test set, resulting in totally eight compounds in the test set.

The training and test sets of agonists and antagonists were docked into their respective crystal structures (PDB ID: 4MR7 for antagonists and 4MS4 for agonists) [65] before a Truncated Newton minimization sampling was performed with the maximum number of sampling steps set to 1000 (default settings). The flexible region of the receptor included the amino acids in the binding pocket. A similar sampling minimization procedure was also performed for the unbound ligand and receptor. The sampling simulations were performed in an implicit water model (default settings) [36,37]. The three necessary energy descriptors (van der Waals, electrostatic and a cavity term energies of bound and unbound states), were calculated from the simulations of the training sets. Together with the experimentally obtained activity values these energy values were used to derive the coefficients α, β, and γ, and for making linear regression models and statistical evaluation by comparing the predicted pIC_50_ values of the test set to the provided experimental values. The models were also applied to the false positive agonists retrieved by the docking protocol, to evaluate the predictability of the models.

## 5. Conclusions

The low number of available active ligands towards the GABA_B_-R complicates and limits the use of both ligand-based and structure-based approaches. The quality of ligand-based methods and validation of the predictability of structure-based models are dependent on both the number and diversity of active ligands. Fingerprinting methods were used and evaluated, but did not give reliable results. The pharmacophore models combined with docking on the other hand, showed a discrimination between actives and DUD-E decoys acceptable for a VS process. The pharmacophore mapping gave false positives, but docking reduced this number. The present study indicates that the use of LIA models only slightly will affect the outcome of a VS campaign as only one DUD-E agonist decoy from docking was recognised as a false positive by the agonist LIA model. The structural analysis of X-ray structure complexes and docking showed that certain LB1 interactions are necessary for anchoring the ligands in the crevice of the VFT, and that the interactions with residues of LB2 will impact the function of the ligand and the affinity. On the background of previously mentioned studies and in light of the results in this study, there is a strong correlation with the specific ligand features and the number of interactions with key residues in both LB1 and LB2. In circumstances where a low number of actives is known, exhaustive structure-based methods in combination with pharmacophore modelling may lead to identification of novel compounds.

## Figures and Tables

**Table 1:** The pharmacophore hypotheses with the number of active compounds (#Actives) in the cluster, the number of true positives (TP), false negatives (FN), false positives (FP) and true negatives (TN) obtained after mapping actives and DUD-E decoys to the pharmacophore model. These values were used to calculate the Matthews correlation coefficient (MC) and Goodness of Hit (GH). AR: number of actives retrieved after mapping all active compounds to the models. Abbreviations; Ant: antagonists, Ago: agonists. Feature abbreviations; hydrophobic feature: H, hydrogen bond acceptor feature: A, hydrogen bond donor feature: D, aromatic feature: R, positively charged feature: P, and negatively charged feature: N.

Cluster	Hypothesis	#Actives	Actives		Decoys		MCC	GH	AR	
			TP	FN	FP	TN			Ant	Ago
1	AHHHR *	6	3	3	21	279	0.22	0.20	1	2
2	ADN	12	7	5	30	270	0.31	0.27	11	11
3	ANPR	11	9	2	2	548	0.82	0.82	9	0
4	NPR	9	9	0	2	448	0.90	0.95	12	13
5	DDDHR	4	4	0	55	145	0.22	0.22	3	11
6	DDDN	13	13	0	9	641	0.76	0.68	7	40

*: Outliers (both agonists and antagonists).

**Table 2:** The statistical values and LIA parameters (α, β and γ) of the LIA models for agonists and antagonists.

LIA Model	R^2^	Standard Deviation	*p*	α	β	γ
Agonist	0.61	0.322	0.074	−0.015	−0.0012	0.34
Antagonist	0.98	0.41	0.00445	−0.1707	0.0073	−0.842

## Supplementary Materials

The following are available online, Table S1: Biological data. The structure and activity of the 55 agonists and antagonist in the set of active GABA_B_-R compounds. Table S2: structure of inactive compounds.
