# Supplementary material for: In Silico Methods for the Discovery of Orthosteric GABAB Receptor Compounds
Source: Molecules. 2019 Mar 7;24(5):935. doi: 10.3390/molecules24050935 (PMC6429233; doi:10.3390/molecules24050935)
Supplement: Supplementary file 1 [file molecules-24-00935-s001.pdf]

molecules

Supplementary Material

In Silico Methods for the Discovery of Orthosteric $GABA_B$ Receptor Compounds

Linn M. Evenseth [1], Dawid Warszycki [2], Andrzej J. Bojarski [2], Mari Gabrielsen [1] and Ingebrigt Sylte [1,*]

[1] Molecular Pharmacology and Toxicology, Department of Medical Biology, Faculty of Health Sciences, UiT—The Arctic University of Norway, NO-9037 Tromsø, Norway; linn.evenseth@uit.no (L.M.E.); mari.gabrielsen@uit.no (M.G.)
[2] Department of Medicinal Chemistry, Institute of Pharmacology, Polish Academy of Science, Smetna 12, 31-343 Kraków, Poland; warszyc@if-pan.krakow.pl (D.W.); bojarski@if-pan.krakow.pl (A.J.B.)
* Correspondence: ingebrigt.sylte@uit.no; Tel.: +47-77-64-4705

Table S1 - **Biological data.** The table shows the dataset of active $GABA_B$-R compounds. Threshold values for being including in the set of active compounds were: IC_{50} < 4100 nM, K_i < 1500 nM, EC_{50} < 25 µM, or fold changes/inhibition indicating higher activity than GABA. Compounds were structurally clustered into six clusters based on fingerprints. The compounds are shown with activity values and the source of the data (reference). Cluster 1: four agonists and two antagonists. Cluster 2: 12 agonists. Cluster 3: 11 antagonists. Cluster 4: nine agonists. Cluster 5: four agonists. Cluster 6: 13 agonists.

Compound		Activity		Source
Structure	Name	Type	Value	
		Cluster 1		
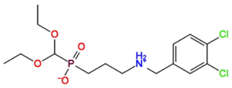	CGP52432*	IC_{50}	0.055 µM	[1]
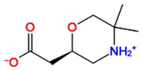	SCH50911	IC_{50}	1.100 µM	[2]
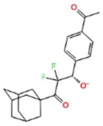	CHEMBL 2322934 (S)	EC_{50}	24.90 µM	[3]
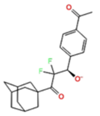	CHEMBL 2322934 (R)	EC_{50}	24.90 µM	[3]

Table S1. *Cont.*

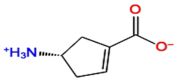	CHEMBL239812 1 (S)	Max. response	75.3%	[4]
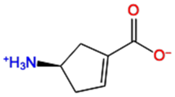	CHEMBL239812 1 (R)	Max. response	75.3%	[4]

1. *GABA B receptor pharmacology: a tribute to Norman Bowery*; Blackburn, T.P., Bowery, N., Eds.; Advances in Pharmacology; Elsevier, Acad. Press: Amsterdam, The Netherlands, 2010; ISBN 978-0-12-378647-0. Inhibition of binding of [^{3}H]CGP27492
2. Bolser, D.C.; Blythin, D.J.; Chapman, R.W.; Egan, R.W.; Hey, J.A.; Rizzo, C.; Kuo, S.C.; Kreutner, W. The pharmacology of SCH 50911: a novel, orally-active GABA-beta receptor antagonist. *J. Pharmacol. Exp. Ther.* **1995**, *274*, 1393–1398. Inhibition of the binding of 5 nM [3H]GABA.
3. Han, C.; Salyer, A.E.; Kim, E.H.; Jiang, X.; Jarrard, R.E.; Powers, M.S.; Kirchhoff, A.M.; Salvador, T.K.; Chester, J.A.; Hockerman, G.H.; et al. Evaluation of Difluoromethyl Ketones as Agonists of the γ-Aminobutyric Acid Type B (GABA B Receptor). *J. Med. Chem.* **2013**, *56*, 2456–2465. Inhibition of forskolin stimulated (10 μM) cAMP production.
4. Locock, K.E.S.; Yamamoto, I.; Tran, P.; Hanrahan, J.R.; Chebib, M.; Johnston, G.A.R.; Allan, R.D. γ-Aminobutyric Acid(C) (GABA C) Selective Antagonists Derived from the Bioisosteric Modification of 4-Aminocyclopent-1-enecarboxylic Acid: Amides and Hydroxamates. *J. Med. Chem.* **2013**, *56*, 5626–5630. Percent of maximum GABA (300 μM) response at a concentration of 300 μM of tested compound.

Cluster 2

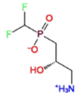	56 (R)$^+$	IC_{50}	0.213 μM	[5]
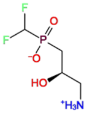	56 (S)	IC_{50}	0.213 μM	[5]
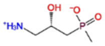	55 (R)	IC_{50}	0.077 μM	[5]
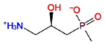	55 (S)	IC_{50}	0.077 μM	[5]
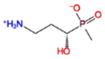	52 (S)	IC_{50}	1.160 μM	[5]

Table S1. *Cont.*

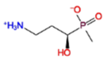	52 (R) +	IC_{50}	1.160 μM	[5]
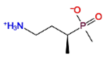	45 (S)	IC_{50}	0.140 μM	[5]
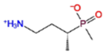	45 (R)	IC_{50}	0.140 μM	[5]
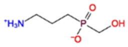	44	IC_{50}	1.050 μM	[5]
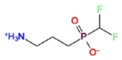	43+	IC_{50}	0.089 μM	[5]
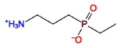	ChEMBL325921	IC_{50}	1.350 μM (*)	[5]
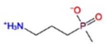	ChEMBL112710	IC_{50}	0.0066 μM (*)	[5]

5. Froestl, W.; Mickel, S.J.; Hall, R.G.; von Sprecher, G.; Strub, D.; Baumann, P.A.; Brugger, F.; Gentsch, C.; Jaekel, J. Phosphinic Acid Analogs of GABA. 1. New Potent and Selective GABAB Agonists. *J. Med. Chem.* **1995**, *38*, 3297–3312.: Inhibition of binding of 10nM [^{3}H]Baclofen (*) or 2 nM [3H]CGP 27492 to $GABA_B$ receptors from rat cortex.

Cluster 3

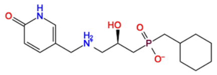	CGP63360	IC_{50}	0.0390 μM	[1]
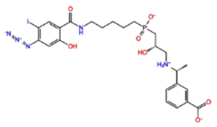	CGP71782	IC_{50}	0.0024 μM	[6]
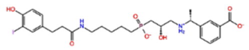	CGP64213*	IC_{50}	0.002 μM	[6]
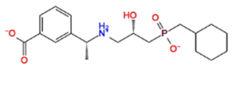	CGP56999	IC_{50}	0.0004 μM	[6]
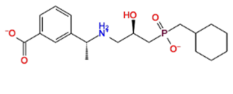	CGP56433	IC_{50}	0.080 μM	[1]

Table S1. *Cont.*

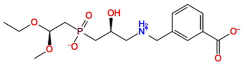	CGP61334	IC_{50}	0.036 µM	[1]
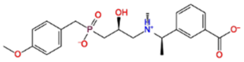	CGP62349(S)	IC_{50}	0.002 µM	[1]
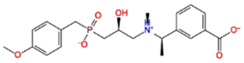	CGP62349(R)	IC_{50}	0.002 µM	[1]
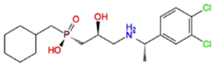	CGP54626(S)	IC_{50}	0.002 µM	[6]
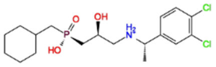	CGP54626(R)	IC_{50}	0.002 µM	[6]
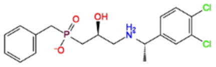	CGP55845*	IC_{50}	0.006 µM	[1]

1. *GABA B receptor pharmacology: a tribute to Norman Bowery*; Blackburn, T.P., Bowery, N., Eds.; Advances in pharmacology; Elsevier, Academic Press: Amsterdam, The Netherlands, 2010; ISBN 978-0-12-378647-0. Inhibition of binding of [3H]CGP27492
6. Kaupmann, K.; Huggel, K.; Heid, J.; Flor, P.J.; Bischoff, S.; Mickel, S.J.; McMaster, G.; Angst, C.; Bittiger, H.; Froestl, W.; et al. Expression cloning of GABA(B) receptors uncovers similarity to metabotropic glutamate receptors. *Nature* **1997**, *386*, 239–246. Inhibition of binding of 0.1 nM [125I]CGP64213

Cluster 4

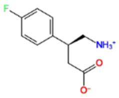	13(S)	IC_{50}	0.360 µM	[5]
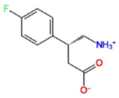	13(R)+	IC_{50}	0.360 µM	[5]
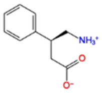	14(S)	IC_{50}	0.880 µM	[5]
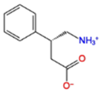	14(R)+	IC_{50}	0.880 µM	[5]
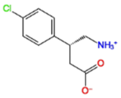	R-Baclofen	IC_{50}	0.015 µM	[5]

Table S1. *Cont.*

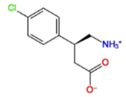	S-Baclofen	IC_{50}	1.770 µM	[5]
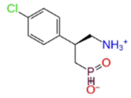	12(S)	IC_{50}	0.039 µM	[5]
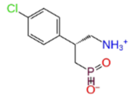	12(R)	IC_{50}	0.039 µM	[5]
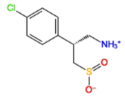	ChEMBL312675	IC_{50}	0.200 µM	[7]

5. Froestl, W.; Mickel, S.J.; Hall, R.G.; von Sprecher, G.; Strub, D.; Baumann, P.A.; Brugger, F.; Gentsch, C.; Jaekel, J. Phosphinic Acid Analogs of GABA. 1. New Potent and Selective GABAB Agonists. *J. Med. Chem.* **1995**, *38*, 3297–3312. Inhibition of binding of the binding 10nM [³H]Baclofen to $GABA_B$ receptor from rat cortex.
7. Carruthers, N.I.; Spitler, J.M.; Shing-Chun Wong; Blythin, D.J.; Xiao Chen; Ho-Jane Shue; Mittelman, S. Synthesis and resolution of β-(aminomethyl)-4-chlorobenzeneethanesulfinic acid a potent gabaB receptor ligand. *Bioorg. Med. Chem. Lett.* **1995**, *5*, 237–240. Not specified

Cluster 5

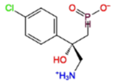	27(S)	IC_{50}	0.065 µM	[5]
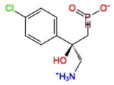	27(R)$^+$	IC_{50}	0.065 µM	[5]
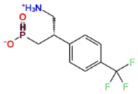	ChEMBL325507(R)	Inhibition	66%	[5]
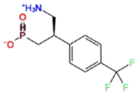	ChEMBL325507(S)	Inhibition	66%	[5]

5. Froestl, W.; Mickel, S.J.; Hall, R.G.; von Sprecher, G.; Strub, D.; Baumann, P.A.; Brugger, F.; Gentsch, C.; Jaekel, J. Phosphinic Acid Analogs of GABA. 1. New Potent and Selective GABAB Agonists. *J. Med. Chem.* **1995**, *38*, 3297–3312. Inhibition of binding of 10nM [³H]Baclofen

Cluster 6

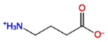	GABA	IC_{50}	0.025 µM	[5]

Table S1. *Cont.*

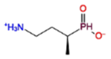	7(S)	IC_{50}	0.920 µM	[5]
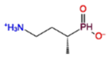	7(R)$^{+}$	IC_{50}	0.920 µM	[5]
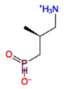	8(S)	IC_{50}	0.780 µM	[5]
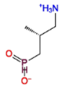	8(R)$^{+}$	IC_{50}	0.780 µM	[5]
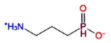	ChEMBL112203	IC_{50}	0.0024 µM	[5]
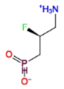	ChEMBL448343	K_i	0.0051 µM	[8]
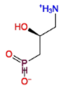	16(S)	IC_{50}	0.018 µM	[5]
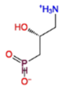	16(R)	IC_{50}	0.018 µM	[5]
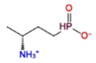	9(S)	IC_{50}	0.5 µM	[5]
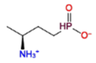	9(R^{+})	IC_{50}	0.5 µM	[5]
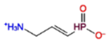	29^{+}	IC_{50}	0.280 µM	[5]
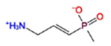	68^{+}	IC_{50}	0.665 µM (*)	[5]

Table S1. *Cont.*

5. Froestl, W.; Mickel, S.J.; Hall, R.G.; von Sprecher, G.; Strub, D.; Baumann, P.A.; Brugger, F.; Gentsch, C.; Jaekel, J. Phosphinic Acid Analogs of GABA. 1. New Potent and Selective GABAB Agonists. *J. Med. Chem.* **1995**, *38*, 3297–3312. Inhibition of binding of 10nM [3H]Baclofen or 2 nM [3H]CGP 27492 (*) to $GABA_B$ receptors from rat cortex.
8. Alstermark, C.; Amin, K.; Dinn, S.R.; Elebring, T.; Fjellström, O.; Fitzpatrick, K.; Geiss, W.B.; Gottfries, J.; Guzzo, P.R.; Harding, J.P.; et al. Synthesis and Pharmacological Evaluation of Novel γ-Aminobutyric Acid Type B (GABAB) Receptor Agonists as Gastroesophageal Reflux Inhibitors. *J. Med. Chem.* **2008**, *51*, 4315–4320. Displacement of [3H]GABA.

Low affinity antagonists added for LIA modelling

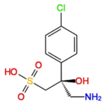	2-hydroxy-saclofen	pIC50	4.1M	Guidetopharmacology.org
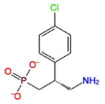	phaclofen	pIC50	4.1M	Guidetopharmacology.org
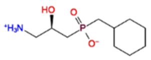	CGP51776	IC50	6 µM	[1]
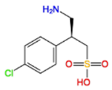	saclofen	pIC50	3.5M	Guidetopharmacology.org

1. *GABA B receptor pharmacology: a tribute to Norman Bowery*; Blackburn, T.P., Bowery, N., Eds.; Advances in pharmacology; Elsevier, Academic Press: Amsterdam, The Netherlands 2010; ISBN 978-0-12-378647-0. Inhibition of binding of [3H]CGP27492

+Agonists included in the test set used to calculate the LIA coefficients

*Antagonists included in the test set used to calculate the LIA coefficients

Table S2 - **Inactive compounds.** The structure of the inactive compounds. The dataset contains totally inactive and low affinity/activity GABAB-R compounds.

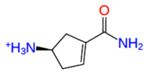
title: C6H10N2O

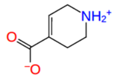
title: C6H9NO2

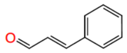
title: C9H8O

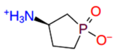
title: C4H10NO2P

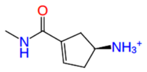
title: C7H12N2O

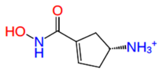
title: C6H10N2O2

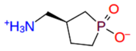
title: C5H12NO2P

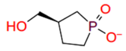
title: C5H11O3P

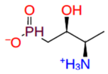
title: C4H11NO3P

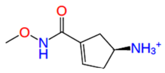
title: C7H12N2O2

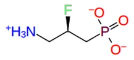
title: C3H9FNO3P

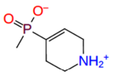
title: C6H12NO2P

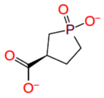
title: C5H9O4P

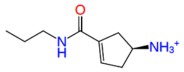
title: C9H16N2O

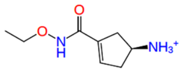
title: C8H14N2O2

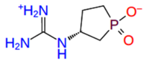
title: C5H12N3O2P

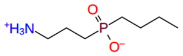
title: C7H18NO2P

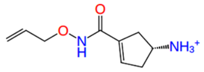
title: C9H14N2O2

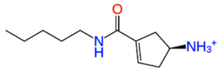
title: C11H20N2O

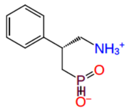
title: C9H13NO2P

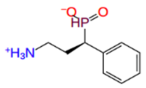
title: C9H13NO2P

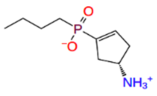
title: C9H18NO2P

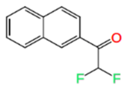
title: C12H8F2O

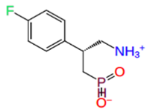
title: C9H12FNO2P

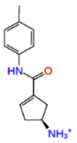
title: C13H16N2O

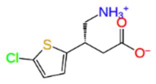
title: C8H10ClNO2S

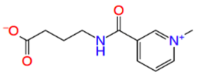
title: C12H18N2O7S

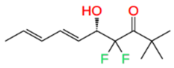
title: C12H18F2O2

Table S2. *Cont.*

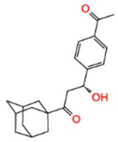

title: C21H26O3

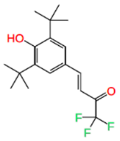

title: C18H23F3O2

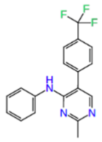

title: C18H14F3N3

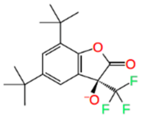

title: C17H21F3O3

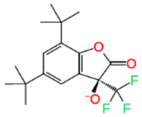

title: C17H21F3O3

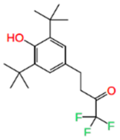

title: C18H25F3O2

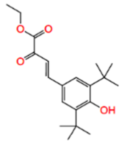

title: C20H28O4

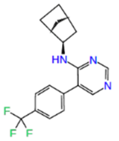

title: C18H18F3N3

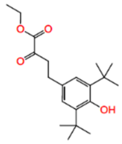

title: C20H30O4

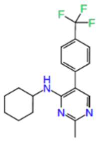

title: C18H20F3N3

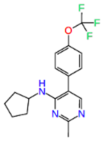

title: C17H18F3N3O

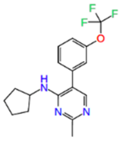

title: C17H18F3N3O

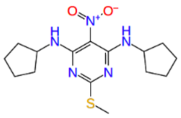

title: C15H23N5O2S

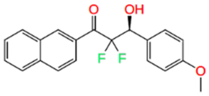

title: C20H16F2O3

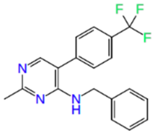

title: C19H16F3N3

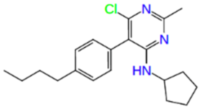

title: C20H26ClN3

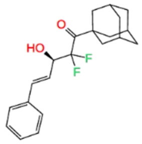

title: C21H24F2O2

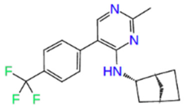

title: C19H20F3N3

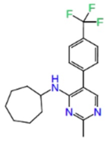

title: C19H22F3N3

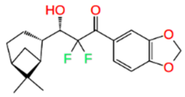

title: C19H22F2O4

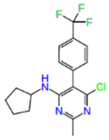

title: C17H17ClF3N3

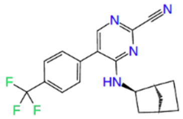

title: C19H17F3N4

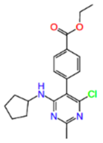

title: C19H22ClN3O2

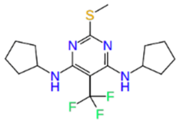

title: C16H23F3N4S

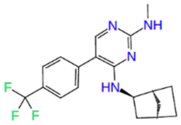

title: C19H21F3N4

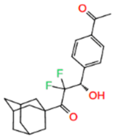

title: C21H24F2O3

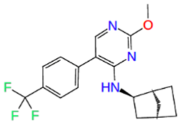

title: C19H20F3N3O

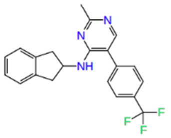

title: C21H18F3N3

Table S2. *Cont.*

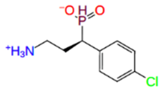

title: C9H12ClNO2P

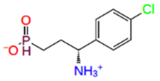

title: C9H12ClNO2P

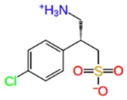

title: C9H12ClNO3S

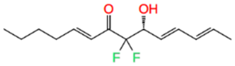

title: C14H20F2O2

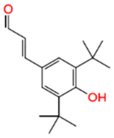

title: C17H24O2

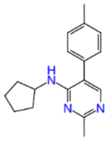

title: C17H21N3

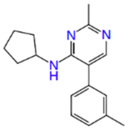

title: C17H21N3

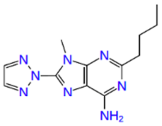

title: C12H16N8

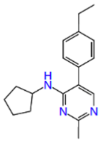

title: C18H23N3

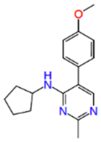

title: C17H21N3O

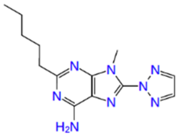

title: C13H18N8

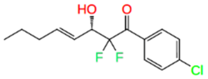

title: C14H15ClF2O2

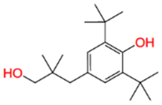

title: C19H32O2

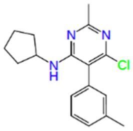

title: C17H20ClN3

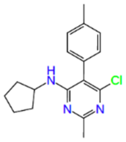

title: C17H20ClN3

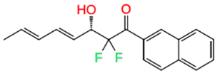

title: C18H16F2O2

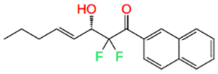

title: C18H18F2O2

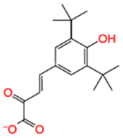

title: C18H24O4

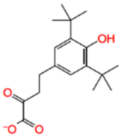

title: C18H26O4

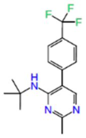

title: C16H18F3N3

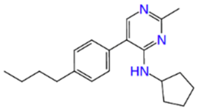

title: C20H27N3

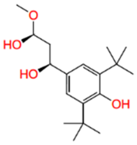

title: C18H30O4

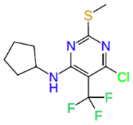

title: C11H13ClF3N3S

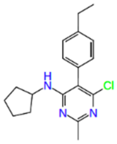

title: C18H22ClN3

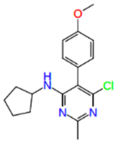

title: C17H20ClN3O

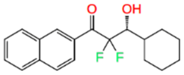

title: C19H20F2O2

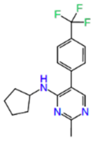

title: C17H18F3N3

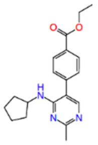

title: C19H23N3O2

Table S2. *Cont.*

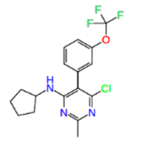

title: C17H17ClF3N3O

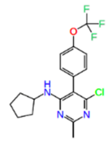

title: C17H17ClF3N3O

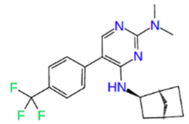

title: C20H23F3N4

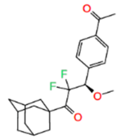

title: C22H26F2O3

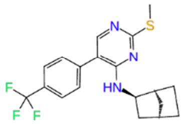

title: C19H20F3N3S

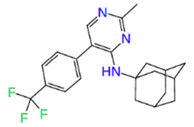

title: C22H24F3N3

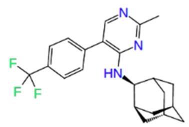

title: C22H24F3N3

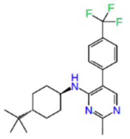

title: C22H28F3N3

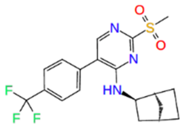

title: C19H20F3N3O2S

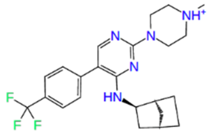

title: C23H28F3N5

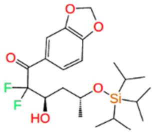

title: C22H34F2O5Si

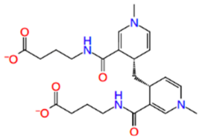

title: C23H30N4Na2O6

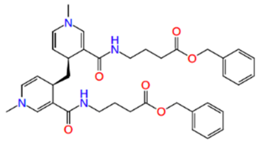

title: C37H44N4O6
